# Pembrolizumab-induced secondary adrenal insufficiency due to adrenocorticotrophic hormone deficiency in a patient with non-small-cell lung carcinoma: a case report

**DOI:** 10.1186/s40780-024-00332-2

**Published:** 2024-02-16

**Authors:** Tatsuhiro Fujimiya, Kanako Azuma, Yuki Togashi, Koji Kuwata, Sakae Unezaki, Hironori Takeuchi

**Affiliations:** 1 Department of Practical Pharmacy, School of Pharmacy, Tokyo University of Pharmacy and Life Sciences, 1432-1, Horinouchi, 192-0392 Hachioji-city, Tokyo, Japan; 2 Department of Pharmacy, Tokyo Medical University Hospital, 6-7-1, Nishi-shinjuku, Shinjuku-ku, 160-0023 Tokyo, Japan; 3 Department of Respiratory Medicine, Tokyo Medical University Hospital, 6-7-1, Nishi-shinjuku, Shinjuku-ku, 160-0023 Tokyo, Japan; 4 Department of Diabetes, Metabolism and Endocrinology, Tokyo Medical University Hospital, 6-7-1, Nishi-shinjuku, Shinjuku-ku, 160-0023 Tokyo, Japan

**Keywords:** Pembrolizumab, Secondary adrenal insufficiency, Immune-related adverse events, Non-small-cell lung carcinoma, Case report

## Abstract

**Background:**

Pembrolizumab can cause immune-related adverse events such as adrenal insufficiency (AI). However, there is no consensus regarding appropriate monitoring of adrenal function during subsequent chemotherapy in patients who have received immune checkpoint inhibitors (ICIs) such as pembrolizumab.

**Case presentation:**

In this report, we discuss the case of a 60s-year-old male patient with non-small cell lung cancer receiving chemotherapy who developed secondary AI due to adrenocorticotrophic hormone (ACTH) deficiency 8 months after the discontinuation of pembrolizumab, which was 17 months after the initiation of pembrolizumab immunotherapy. After 5 months of chemotherapy, he developed fever and diarrhoea, after which chemotherapy was discontinued. Thereafter, he was hospitalised owing to the development of general fatigue and anorexia. Although cortisol and ACTH levels were not measured during chemotherapy, they were measured before hospitalisation, and secondary AI was suspected. After admission, a detailed endocrine workup was performed, and the patient was diagnosed with secondary AI due to ACTH deficiency. Treatment with hydrocortisone was initiated, which markedly improved his general fatigue and anorexia. The patient showed no evidence of progressive disease 9 months after the discontinuation of pembrolizumab.

**Conclusions:**

Although rare, the possibility of AI should be considered in patients who have received ICIs when nonspecific symptoms develop during or after subsequent chemotherapy, and measurements of endocrine function (including cortisol and ACTH levels) should be performed.

## Background

Pembrolizumab, an immune checkpoint inhibitor (ICI) that exhibits antitumour effects by inhibiting programmed cell death 1 (PD-1), has been approved for the treatment of malignant melanoma, non-small-cell lung cancer (NSCLC), urothelial carcinoma, renal cell carcinoma, and other types of cancer. Numerous clinical trials of ICIs are currently underway, and their use in various cancer types is expected to increase in the future. However, ICIs can cause immune-related adverse events (irAEs), the most common of which is hypothyroidism [1].

Hypophysitis is a rare irAE caused by anti-PD-1 antibodies, occurring in less than 1% of patients [2, 3]. Hypophysitis associated with ICIs causes secondary adrenal insufficiency (AI) and hypopituitarism. Given that hypophysitis can be fatal if not properly treated, early detection and initiation of steroid treatment are imperative [4]. Adrenocorticotropic hormone (ACTH) deficiency is a form of hypopituitarism presenting symptoms of secondary AI. Since the symptoms of secondary AI due to ACTH deficiency are non-specific, endocrine function tests, including measurements of cortisol and ACTH levels, are necessary for diagnosis. However, how AI should be monitored following the discontinuation of pembrolizumab administration remains controversial.

In this report, we present a case in which the patient developed secondary AI due to ACTH deficiency 17 months after the initiation of pembrolizumab for NSCLC, which occurred 8 months after the last administration of pembrolizumab. In this case, it was difficult to determine whether the non-specific symptoms he experienced during and after chemotherapy were caused by AI. This case report was prepared following the CARE guidelines [5].

## Case presentation

In July 20XX-5, a 60s-year-old man was diagnosed with stage IIIB right upper lobe lung adenocarcinoma (cT4N2M0). A partial response was achieved after two cycles of cisplatin and S-1 chemotherapy with concurrent radiotherapy (66 Gy/33 fr). Subsequently, two cycles of additional chemotherapy were administered, resulting in a complete response. In May 20XX-2, computed tomography (CT) showed a nodule in the right lung base, and in July 20XX-2, positron emission tomography-CT (PET-CT) showed areas of accumulation in the small intestine and liver. In August 20XX-2, small bowel endoscopy revealed a type II tumour in the small intestine, and a biopsy revealed this to be a thyroid transcription factor-1 (TTF-1)-positive adenocarcinoma, which was diagnosed as a recurrence of NSCLC. At that time, programmed cell death 1 ligand 1 (PD-L1) expression was 100%, although findings were negative for epidermal growth factor receptor (EGFR) mutations (exon18 G719X, exon19 deletion, exon20 S768I, exon20 insertion, exon20 T790M, exon21 L858R, and exon21 L861Q), anaplastic lymphoma kinase (ALK) fusion, and c-ros oncogene 1 (ROS1) fusion. The patient had no significant history of complications or allergies. In September 20XX-2, treatment with 200 mg of pembrolizumab was initiated. Nine months before the visit (June 20XX-1; day 22 of the 11^th^ cycle of pembrolizumab), CT revealed increased liver metastasis, and pembrolizumab was discontinued. Subsequent chemotherapy with carboplatin (CBDCA) and nanoparticle albumin-bound paclitaxel (nab-PTX) was initiated. During the third cycle of chemotherapy (August 20XX-1), the patient began to experience fatigue and dyspnoea. Anorexia was not persistent. At the end of the fourth cycle, CT showed a reduction in the size of the liver lesion. At the beginning of the fifth cycle of chemotherapy (November 20XX-1), the patient reported shortness of breath upon exertion. Subsequently, fatigue and anorexia became persistent. His D-dimer level was 1.13 µg/mL; however, no evidence was observed for pulmonary embolism on contrast-enhanced CT. CT showed no metastasis in the adrenal gland, and follow-up was considered appropriate. On December 20XX-1, day 23 of the fifth cycle of chemotherapy (eight days after the last dose of nab-PTX), the patient presented with fever and diarrhoea, and Common Terminology Criteria for Adverse Events Version 5.0 (CTCAE v5.0) grade 1 neutropenia was observed. As the patient was undergoing chemotherapy, treatment with levofloxacin hydrate was initiated. On day 26 (11 days after the last dose of nab-PTX), diarrhoea persisted; therefore, levofloxacin hydrate was discontinued, and the patient was treated with antipyretic agents, anti-flatulent agents, and fluid replacement. Diarrhoea stopped on day 35 (20 days after the last dose of nab-PTX); however, his fatigue persisted. On the same day, treatment with inhaled budesonide/formoterol was initiated to alleviate persistent dyspnoea. The patient continued to experience fatigue and anorexia, and the patient’s Eastern Cooperative Oncology Group performance status (ECOG-PS) was 2, which raised concerns about continuing with chemotherapy. In January 20XX (64 days after the last dose of nab-PTX), fatigue persisted, and head magnetic resonance imaging (MRI) showed no signs of brain metastasis.

In February 20XX (92 days after the last dose of nab-PTX), the patient visited the hospital because he had been experiencing fatigue and anorexia for more than 3 months during chemotherapy. Upon presentation to the hospital, his CTCAE v5.0 grades for fatigue and anorexia were 2 and 3, respectively. Cortisol and ACTH levels were normal during pembrolizumab administration (Table 1). Three months had passed since the last dose of chemotherapy, and irAEs were suspected given the patient’s history of pembrolizumab administration. The patient was receiving levothyroxine sodium hydrate for pembrolizumab-induced hypothyroidism. The cortisol and ACTH levels were not measured during chemotherapy; however, these values were markedly decreased (cortisol: 0.3 µg/dL; ACTH < 2.0 pg/mL) when his levels were tested at the hospital prior to admission, and secondary AI was suspected. Other laboratory tests revealed that serum potassium (3.3 mmol/L), serum creatinine (1.41 mg/dL), and C-reactive protein (CRP; 1.00 mg/dL) levels were outside the reference range. As he had difficulty walking, emergency admission was recommended; however, the patient preferred to return to his home temporarily. Treatment with 100 mg of intravenous hydrocortisone sodium succinate and 30 mg/day of oral hydrocortisone was administered on an outpatient basis and the patient returned to his home.

**Table 1:** Cortisol and ACTH levels from before initiation to after discontinuation of pembrolizumab

Cycle of pembrolizumab	Before	2^nd^	3^rd^	4^th^	5^th^	6^th^	7^th^	8^th^	9^th^	10^th^	after 11^th^
Cortisol, µg/dL (Reference range: 6.2–18.0)	–	–	7.4	11.4	7.5	9.5	8.9	–	8.4	9.7	8.1
ACTH, pg/mL (Reference range: 7.2–63.3)	19.9	–	51.9	55.8	26.6	32.4	33.5	–	25.1	47.6	48.2

ACTH: adrenocorticotropic hormone

In March 20XX, the patient was admitted to the hospital (three days after his outpatient visit). He reported weight loss of approximately 15 kg over the past 4 months, stating that he could hardly eat. However, following an initial administration of hydrocortisone, the patient’s ECOG-PS improved, and he could walk upon admission. Vital signs on admission were as follows: temperature, 37.1 °C; blood pressure, 120/64 mmHg; pulse rate, 105 beats/min. No other specific physical findings were identified. The medications used at the time of admission were levothyroxine sodium hydrate (75 µg/day), bilastine (20 mg/day), zolpidem tartrate (5 mg/day), an anti-flatulent agent, Rikkunshito, inhaled budesonide/formoterol, and hydrocortisone (30 mg/day). On the second day after admission, contrast-enhanced pituitary MRI showed no abnormal findings; no pituitary nodules or pituitary stalk thickening were noted, and a preserved high signal in the posterior lobe on T1WI was observed with no meningeal thickening. Levels of cortisol (0.3 µg/dL), ACTH (< 2.0 pg/mL), luteinising hormone (LH; 9.9 mIU/mL), follicle-stimulating hormone (FSH; 19.9 mIU/mL), prolactin (PRL; 52.9 ng/mL), growth hormone (GH; 1.34 ng/mL), arginine vasopressin (4.9 pg/mL), and somatomedin C (69 ng/mL) were measured. Cortisol and ACTH levels were low from the first to the second day of admission. On the third day, cortisol was hyporesponsive (peak level < 18 µg/dL) in a rapid ACTH loading test (Table 2A). On the fourth day, both cortisol and ACTH levels exhibited hyporesponsiveness, as did thyroid-stimulating hormone (TSH) levels (peak level < 6 µIU/mL) during a loading test involving the administration of corticotropin-releasing hormone (CRH), thyrotropin-releasing hormone (TRH), and gonadotropin-releasing hormone (GnRH; Table 2B). On the fifth day, a normal GH response was observed in the growth hormone-releasing peptide-2 (GHRP-2) loading test (Table 2C). Based on these examination findings, the patient was diagnosed with grade 3 secondary AI (CTCAE v5.0) due to ACTH deficiency. The onset of secondary AI was approximately 17 months after starting pembrolizumab and eight months after pembrolizumab discontinuation. Based on the patient’s fever and diarrhoea after five cycles of chemotherapy, the physician suspected viral enteritis. Although infection may have been the trigger for AI, the fever and diarrhoea had improved with treatment, and the patient’s fatigue and anorexia persisted until three days before admission. The physician suspected irAEs were more likely based on the low ACTH level and the patient’s treatment history of pembrolizumab. Adrenal, pituitary, or hypothalamus diseases other than irAEs could not be completely ruled out, though the physician ruled out tumour-related AI as no abnormal findings were obtained on the abdominal CT and head MRI prior to admission and pituitary MRI during admission. As of three days before admission, other than pembrolizumab, the patient was not on any other medications known to induce AI.

**Table 2A:** Rapid ACTH loading test on the third day after admission

	Time (min)		
	Basal	30	60
Cortisol, µg/dL	0.2	1.2	1.4

Tetracosactide acetate (0.25 mg) was injected intravenously. ACTH: adrenocorticotropic hormone

**Table 2B:** CRH/TRH/GnRH loading test on the fourth day after admission

			Time (min)			
		Reference range*	Basal	30	60	90
Cortisol	µg/dL	(6.2–18.0)	0.3	0.3	0.3	0.3
ACTH	pg/mL	(7.2–63.3)	< 2.0	< 2.0	< 2.0	< 2.0
TSH	µIU/mL	(0.50–5.00)	0.58	4.29	4.19	–
PRL	ng/mL	(4.0–14.0)	11.4	79.0	67.4	–
LH	µg/dL	(2.2–8.4)	9.5	35.7	41.9	39.2
FSH	mIU/mL	(1.8–12.0)	18.7	27.8	31.1	33.1

Corticorelin (100 µg), protirelin (200 µg), and gonadorelin acetate (100 µg) were injected intravenously. ACTH: adrenocorticotropic hormone; CRH: corticotropin-releasing hormone; FSH: follicle-stimulating hormone; GnRH: gonadotropin-releasing hormone; LH: luteinising hormone; PRL: prolactin; TRH: thyrotropin-releasing hormone; TSH: thyroid-stimulating hormone

*Basal levels

**Table 2C:** GHRP-2 loading test on the fifth day after admission

			Time (min)				
		Reference Range*	Basal	15	30	45	60
GH	ng/mL	(< 2.47)	0.62	35.9	34.4	28.5	19.9

Pralmorelin hydrochloride (100 µg) was injected intravenously. GH: growth hormone; GHRP-2: Growth hormone-releasing peptide-2

*Basal levels

The case timeline is summarised in Table 3.

**Table 3:** Case timeline

Date	Events
July 20XX-5	• The patient was diagnosed with stage IIIB lung adenocarcinoma.
July–November 20XX-5	• After chemoradiotherapy, the patient’s lung cancer achieved a complete response.
August 20XX-2	• A recurrence of NSCLC was diagnosed.
September 20XX-2	• Pembrolizumab was started.
June 20XX-1	• After the 11^th^ cycle of pembrolizumab, CT revealed increased liver metastasis, and pembrolizumab was discontinued. CBDCA and nab-PTX chemotherapy were started.
August 20XX-1	• During the third cycle of chemotherapy, the patient began to experience fatigue and dyspnoea. Anorexia was not persistent.
November 20XX-1	• At the beginning of the fifth cycle, the patient reported shortness of breath upon exertion. Fatigue and anorexia became persistent.
December 20XX-1	• On day 23 of the fifth cycle, fever and diarrhoea appeared.
January 20XX	• Fatigue and anorexia persisted without chemotherapy, and the ECOG-PS was 2. It was decided that chemotherapy should be discontinued.
February 20XX	• Cortisol and ACTH levels were markedly decreased, and secondary AI was suspected. Hydrocortisone sodium succinate was intravenously administered, and hydrocortisone was administered orally.
March 20XX: Day 1 of admission	• Cortisol and ACTH levels at 15:00 and 23:00 were low.
Day 2	• Cortisol and ACTH levels at 7:00 and 10:00 were low. The patient’s pituitary was scanned using contrast-enhanced MRI, and basic hormone levels were measured.
Day 3	• Cortisol level was hyporesponsive in a rapid ACTH loading test.
Day 4	• Cortisol, ACTH, and TSH levels were hyporesponsive in the CRH, TRH, and GnRH loading tests.
Day 5	• The GHRP-2 loading test showed a normal response for GH. Based on previous endocrine function tests, the patient was diagnosed with secondary AI due to ACTH deficiency.

ACTH: adrenocorticotrophic hormone; AI: adrenal insufficiency; CBDCA: carboplatin; CRH: corticotropin-releasing hormone; CT: computed tomography; ECOG-PS: Eastern Cooperative Oncology Group performance status; GH: Growth hormone; GHRP-2: growth hormone-releasing peptide-2; GnRH: gonadotropin-releasing hormone; MRI: magnetic resonance imaging; nab-PTX: nanoparticle albumin-bound paclitaxel; NSCLC: non-small-cell lung cancer; TRH: thyrotropin-releasing hormone; TSH: thyroid-stimulating hormone

The patient’s ECOG-PS improved after the start of hydrocortisone treatment. He was discharged after a thorough examination of endocrine function. Hydrocortisone was continued at 15 mg/day, and cortisol and ACTH levels were monitored regularly (Table 4). After hospital discharge, the patient has not experienced any recurrence of symptoms, such as fatigue and anorexia. Since discontinuation of CBDCA and nab-PTX chemotherapy, the patient has been under follow-up for 11 months without any progression of NSCLC.

**Table 4:** Cortisol and ACTH levels during hydrocortisone treatment after hospital discharge

Date of visit in 20XX (day 1 marks the initiation of pembrolizumab administration)	day 535	day 563	day 598	day 633	day 661	day 696	day 724	day 759	day 794
Cortisol, µg/dL (Reference range: 6.2–18.0)	7.9	0.2	13.2	12.4	0.4	8.3	6.0	13.9	5.0
ACTH, pg/mL (Reference range: 7.2–63.3)	< 2.0	< 2.0	< 2.0	< 2.0	< 2.0	< 2.0	< 2.0	< 2.0	< 2.0

ACTH: adrenocorticotropic hormone

## Discussion and conclusions

In this report, we discussed a case of pembrolizumab-induced secondary AI due to ACTH deficiency in a patient presenting with non-specific symptoms, including fatigue and anorexia. To the best of our knowledge, there are no published case reports of secondary AI due to ACTH deficiency diagnosed in patients experiencing non-specific symptoms during or after chemotherapy following the discontinuation of pembrolizumab. The optimal timing of routine monitoring of adrenal function could not be estimated from this case. In addition, the secondary AI observed in this case was not due to isolated ACTH deficiency (IAD) but rather due to ACTH deficiency. Hyposecretion of TSH was also suspected. Thus, it is important to evaluate pituitary function if secondary AI is suspected.

Given that secondary AI can be fatal, early detection and treatment are imperative. In this case, we first measured cortisol and ACTH levels owing to the suspected AI, after which we performed a rapid ACTH loading test. CRH, TRH, and GnRH loading tests, in addition to a GHRP-2 evaluation, further supported the diagnosis of secondary AI due to ACTH deficiency, verifying that these tests are useful for diagnosing secondary AI. Although contrast-enhanced pituitary MRI revealed no abnormal findings, other studies have reported that pituitary findings are often absent in patients with AI caused by anti-PD-1 treatment [6, 7]. Therefore, contrast-enhanced pituitary MRI may be useful for differentiating between AI caused by anti-PD-1 treatment and other pituitary diseases. Steroids are the most common drugs that can result in AI [7]. In this case report, adrenal corticosteroids were administered during chemotherapy; however, since they were only temporarily used as antiemetics, their effect was considered minimal. Weber et al. recommend that all patients receiving ICIs should undergo routine thyroid function tests and other tests for 6 months after completing ICI treatment [8]. However, there is no consensus on how long adrenal function should be routinely monitored during or after chemotherapy following the last administration of pembrolizumab. About 5.32% and 0.42% of ICI-related AI have been reported with the use of anti-cytotoxic T-lymphocyte antigen 4 (CTLA-4) antibody and anti-PD-1 antibody, respectively [3]. To date, several case reports or case series have discussed secondary AI (including IAD) caused by pembrolizumab [9–25]. Among these cases, pembrolizumab-induced secondary AI occurred as early as 1.5 months and as late as 28 months after the initiation of pembrolizumab [16, 24]. The present case was the fourth-latest onset of secondary AI caused by pembrolizumab. The causal relationship between pembrolizumab and secondary AI was determined by applying evidence from individual cases based on the criteria of the Council for International Organizations of Medical Sciences (CIOMS VI, Appendix 7), which revealed that approximately eight criteria were applicable (Table 5) [26]. We considered the likelihood of secondary AI due to pembrolizumab to be high, given the similarity of our case to those described in previous reports and its occurrence in clinical trials.

**Table 5:** Evidence from reported cases (CIOMS VI, Appendix 7)

Evidence from Individual Cases	
Positive rechallenge	No
Definitive (i.e., clearly defined, well documented specific case histories)	Yes
Time to onset plausible	Yes
Positive dechallenge	N/A
Lack of confounding risk factors	Yes
Amount and duration of exposure consistent/plausible with cause and effect	N/A
Corroboration of the accuracy of the case history	Yes
Case clear-cut, easily evaluated	Yes
Co-medication unlikely to play a role	Yes
Investigator’s causality assessment	Yes
Lack of alternative explanation	Yes

IAD is a disorder in which the secretion of ACTH, one of the six anterior pituitary hormones, is impaired [27]. In this case, which was associated with the administration of pembrolizumab, IAD was not diagnosed because of ACTH deficiency and a low TSH response in the TRH loading test (Table 2). It is unclear when the hyposecretion of TSH first occurred in this case. Intercurrent ACTH and TSH deficiency are frequently reported in anti-CTLA-4 treatment-induced hypophysitis [28]. A small number of cases of intercurrent ACTH and TSH deficiency have also been reported in hypophysitis induced by anti-PD-1 treatment [29]. Considering that the diagnostic criteria for ACTH and TSH deficiency differ in these reports, it is important to evaluate both ACTH and the secretory functions of other pituitary hormones. It should be noted that if TSH and ACTH deficiency associated with untreated hypophysitis are found, corticosteroids should be administered before thyroid hormones to prevent precipitating an adrenal crisis [4]. In this case, the adrenal crisis was not critical because levothyroxine sodium hydrate had been administered for pembrolizumab-induced hypothyroidism before the onset of secondary AI. Although studies have reported that hyponatraemia and increased eosinophil count can occur at the onset of secondary AI [6, 7, 15, 25, 30], neither was observed in the current case. Rather, our patient presented with hypothyroidism, which preceded the onset of secondary AI, as in previous reports [12, 13]. It remains unclear whether this observed hypothyroidism is predictive of the onset of secondary AI. Hydrocortisone therapy, a common treatment for AI associated with ICIs [4], was effective for treating secondary AI in our patient. Therefore, if AI is suspected, cortisol and ACTH levels should be measured promptly, the cause should be identified, and steroid therapy should be initiated as soon as possible. It is challenging to differentiate ICI-associated irAEs from chemotherapy-related AEs based on non-specific symptoms reported in our case. When both irAEs and chemotherapy-related AEs are suspected, clinicians should consider the patient’s history of ICI therapy and suspect the possibility of secondary AI, even if symptoms are non-specific.

Despite our thorough examination, our assessment of this case is limited in that it was difficult to determine whether non-specific symptoms occurring during or after chemotherapy are pembrolizumab-associated irAEs or chemotherapy-associated AEs. Advanced malignancies in ICI-treated patients may lead to the symptoms of AI being overlooked in this population. It should be remembered that patients do not always have symptoms of secondary AI, especially in the absence of acute illness. Most cases of pembrolizumab-induced secondary AI occur during treatment with pembrolizumab. Although some cases have been reported to occur after the discontinuation of pembrolizumab [9, 13, 24], there are few reports of secondary AI occurring during or after subsequent chemotherapy. Moreover, further studies and cases need to be reported, as most cases of ICI-induced pituitary function loss have been reported during the administration of ICIs [31]. In contrast, Yamagata et al. reported a case exhibiting many similarities to our case. In their case, AI was detected during treatment with docetaxel and ramucirumab following the discontinuation of pembrolizumab. However, the endocrinologist initiated a follow-up after the diagnosis of hypothyroidism. In addition, regular monitoring of adrenal function and loading tests led to early detection [13]. In our case, the contrast-enhanced pituitary MRI findings were normal and went against the ICI-induced secondary AI to hypohysitis. However, hypophysitis due to ICIs without MRI findings in the pituitary gland has been previously reported [6, 7, 31]. We considered that the absence of MRI findings would not rule out hypophysitis. In the future, the frequency of ICI treatment is expected to increase in patients with various types of malignant tumours. However, there is no standardised protocol concerning the timing and frequency of follow-up for endocrine function after the discontinuation of ICI administration. Additionally, in Japan, insurance claims for the measurement of cortisol and ACTH levels may not be accepted in patients not receiving ICIs because the medical costs are covered by the universal health insurance system. Since ICI-induced secondary AI is much less frequent than hypothyroidism, the adrenal function follow-up method remains controversial. However, the need to address this issue remains important since forms of secondary AI can have a potentially fatal course.

In this case report, the patient presented with non-specific symptoms during and after chemotherapy following the discontinuation of pembrolizumab treatment for NSCLC and was subsequently diagnosed with secondary AI due to ACTH deficiency. Despite increasing reports concerning secondary AI associated with ICI treatment, secondary AI is still rare. More in-depth understanding of ICI-induced secondary AI is required to elucidate the most appropriate strategies for monitoring endocrine function in this patient population.

## Data Availability

All data generated or analysed during this study are included in this published article.

## Abbreviations

ACTH: Adrenocorticotrophic hormone
AI: Adrenal insufficiency
ALK: Anaplastic lymphoma kinase
CBDCA: Carboplatin
CIOMS: the Council for International Organizations of Medical Sciences
CRH: Corticotropin-releasing hormone
CRP: C-reactive protein
CTLA-4: Cytotoxic T-lymphocyte antigen 4
CT: Computed tomography
CTCAE v5.0: Common Terminology Criteria for Adverse Events Version 5.0
ECOG-PS: Eastern Cooperative Oncology Group performance status
EGFR: Epidermal growth factor receptor
FSH: Follicle-stimulating hormone
GHRP-2: Growth hormone-releasing peptide-2
GH: Growth hormone
GnRH: Gonadotropin-releasing hormone
IAD: Isolated adrenocorticotrophic hormone deficiency
ICIs: Immune checkpoint inhibitors
irAEs: Immune-related adverse events
LH: Luteinising hormone
MRI: Magnetic resonance imaging
nab-PTX: Nanoparticle albumin-bound paclitaxel
NSCLC: Non-small-cell lung cancer
PD-1: Programmed cell death 1
PD-L1: Programmed cell death 1 ligand 1
PET-CT: Positron emission tomography-computed tomography
PRL: Prolactin
ROS1: c-Ros oncogene 1
TSH: Thyroid-stimulating hormone
TRH: Thyrotropin-releasing hormone
TTF-1: Thyroid transcription factor-1
